# Novel Bi-Specific Immuno-Modulatory Tribodies Potentiate T Cell Activation and Increase Anti-Tumor Efficacy

**DOI:** 10.3390/ijms23073466

**Published:** 2022-03-23

**Authors:** Margherita Passariello, Asami Yoshioka, Kota Takahashi, Shu-ichi Hashimoto, Rosa Rapuano Lembo, Lorenzo Manna, Koji Nakamura, Claudia De Lorenzo

**Affiliations:** 1 Department of Molecular Medicine and Medical Biotechnology, University of Naples “Federico II”, 80131 Naples, Italy; margherita.passariello@unina.it (M.P.); lorenzo.manna@unina.it (L.M.); 2 Ceinge-Biotecnologie Avanzate s.c.a.r.l., Via Gaetano Salvatore 486, 80145 Naples, Italy; rosa.rapuano@unimi.it; 3 Chiome Bioscience Inc., 3-12-1 Hommachi Shibuya-Ku, Tokyo 151-0071, Japan; asyoshioka@chiome.co.jp (A.Y.); ktakahashi@chiome.co.jp (K.T.); shashimoto@chiome.co.jp (S.-i.H.); knakamura@chiome.co.jp (K.N.); 4 European School of Molecular Medicine, University of Milan, 20122 Milan, Italy

**Keywords:** immunotherapy, cancer, bispecific antibodies, targeted-therapy, intracellular pathways

## Abstract

Cancer immunotherapy has already shown significant improvements by combining different antibodies specific for distinct immune checkpoints, such as Ipilimumab and Nivolumab. Here, we tested combinatorial treatments of immunomodulatory antibodies, previously generated in our laboratory, for their effects on hPBMC activation, either upon stimulation with SEB or in co-cultures with tumor cells by cytokine secretion assays. We found that some of them showed additive or synergistic effects, and on the basis of these observations, we constructed, for the first time, four novel bispecific tribodies (TR), made up of a Fab derived from one anti-IC mAb and two scFvs derived from another mAb targeting a different IC. All four TRs cotargeting either programmed cell death protein 1 (PD-1) and Lymphocyte Activating 3 (LAG-3) or programmed death-ligand 1 (PD-L1) and LAG-3 retained binding affinity for their targets and the antagonistic effects of their parental mAbs, but some of them also showed an increased ability to induce lymphocyte activation and increased in vitro cytotoxicity against tumor cells compared to parental antibodies used either alone or in combinatorial treatments. Furthermore, none of the tribodies showed significant increased cytotoxicity on human cardiomyocytes. Considering that the tribody format reduces production costs (as only one construct provides the inhibitory effects of two antibodies), has an intermediate molecular size (100 kDa) which is well suited for both tumor penetration and an acceptable half-life, we think that these novel immunomodulatory TRBs have the potential to become precious tools for therapeutic applications, particularly in monotherapy-resistant cancer patients.

## 1. Introduction

Immunotherapeutic antibodies have revolutionized cancer treatment over the past three to four decades, demonstrating superior tolerability than conventional cancer treatments such as chemotherapy or radiotherapy, and yielding major improvements in long-term survival [1]. Immune checkpoint inhibitors, such as the anti-Cytotoxic T-Lymphocyte Antigen 4 (CTLA-4) monoclonal antibody (mAb) Ipilimumab, the anti-Programmed Death 1 (PD-1) mAbs Nivolumab and Pembrolizumab, the anti-PD-Ligand 1 (PD-L1) mAbs Atezolizumab and Durvalumab have been approved by FDA for treatment regimens of several malignancies. Other immuno-modulatory mAbs are currently being evaluated in clinical trials, including novel anti-CTLA-4 and anti-PD-1 mAbs, antibodies targeting costimulatory receptors, such as 4-1BB, OX40, CD40, GITR, CD27, and inhibitory immune checkpoints (ICs) such as TIM-3, LAG-3, among others [2].

Immunotherapy, based on monotherapy of antibodies, has obtained some success in cancer patients; however, some failures due to the escape mechanisms of tumor cells through antigen loss/downregulation, have been observed and associated with resistance [3]. With the aim of achieving higher therapeutic indices compared to monotherapy, several preclinical and clinical trials are currently focusing on treatment paradigms employing antibodies in combination with chemotherapy, radiations, targeted drugs, other antibodies, immune checkpoint inhibitors, vaccines, and/or cellular therapies [4]. Combinatorial therapies can also involve multiple immunomodulatory monoclonal antibodies, such as the FDA approved combination of Ipilimumab and Nivolumab for melanoma. Additionally, clinical trials are underway for anti-LAG-3 combined with anti-PD-1 mAb for glioblastoma (NCT02658981) and other cancers (NCT02460224), and the combination of anti-TIM3 and anti-PD-1 antibodies for liver cancer (NCT03680508) and several other solid tumors (NCT03744468) [4,5,6,7,8].

In this scenario, on the basis of the success of these pioneering combinatorial treatments with the aim of tackling the evasion mechanisms adopted by tumors, multispecific immunotherapy is emerging as a second wave of immunotherapeutic compounds which may overcome the limitations of conventional mAbs, and offer advantages in terms of efficacy, safety or both. To date, more than 100 bispecific Antibody (bsAb) formats have been developed and over 50 bsAbs have been investigated in clinical trials [9,10,11,12].

A key structural feature of bsAbs is the presence/absence of the Fc region [11,12,13,14,15,16,17,18]. Fc-free bsAbs display better biodistribution into tumor tissues, higher potency, and less common incidence of immune-related adverse effects (irAEs) [19]; on the other hand, continuous intravenous infusion or structural modifications are needed to prolong their half-lives, such as fusion with polyethylene glycol or human serum albumin [20].

One of the first successful examples of a Fc-free bispecific construct is the anti-CD19/CD3 Bispecific-T cell Engager (BiTE) Blinatumomab, approved in 2014 by the FDA for relapsed/refractory B-cell precursor acute lymphoblastic leukemia (B-ALL) and non-Hodgkin’s Lymphoma [21,22].

Some other types of bsAbs are in the advanced stages of clinical development, such as Glofitamab, an anti-CD20/CD3 construct comprising two Fab regions specific for CD20 and CD3, respectively, currently in a phase I/Ib trial with Obinutuzumab for the treatment of B lymphoma (NCT03075696) [23,24,25]; Pasotuxizumab (BAY 2010112), a T cell engager BiTE targeting prostate-specific membrane antigen (PSMA), in phase I of a clinical trial (NCT01723475) for the treatment of patients with advanced castration-resistant prostate cancer (CRPC) [26].

However, the half-life of Blinatumomab and other BiTEs in humans is about 2 h due to its low molecular weight, i.e., 55 kDa [10,11,27]. Thus, other approaches have been considered, such as the use of tribodies. Tribodies are multifunctional, recombinant antibody derivatives which use the natural in vivo heterodimerization of the heavy chain (Fd fragment) and light chain (L) of a Fab fragment to form a scaffold upon which additional functions can be incorporated with additional binders, such as scFvs. Thus, tribodies are made up of a Fab and two scFvs with a molecular weight (M.W.) of 100 kDa, which allows for a longer half-life in circulation compared to BiTEs [28,29,30,31]. Furthermore, tribodies can combine up to three binding sites in a single molecule, allowing for the creation of either a trispecific monovalent or a bispecific but bivalent construct with respect to bispecific monovalent BiTEs [30,31].

Herein, we investigated the efficacy of novel combinations of human anti-PD-L1, anti-PD-1 and anti-LAG-3 mAbs, previously generated in our laboratory [32,33,34,35,36], in order to choose the most effective combinations. On the basis of these findings, we constructed four novel different bispecific immuno-modulatory tribodies by fusing the Fab portion of one anti-IC antibody with two scFv domains of a different mAb, targeting a distinct IC.

The novel tribodies were expressed, purified, and analyzed for their ability to bind and potentiate T cell activation, in comparison with the parental mAbs or their combinations.

## 2. Results

### 2.1. In Vitro Effects of Combined Immunomodulatory mAbs on the Stimulation of hPBMCs

In order to test the efficacy of different combinations of immunomodulatory antibodies for T cell activation, combinatorial treatments were investigated by using monoclonal antibodies against LAG-3, PD-L1 or PD-1, previously generated in our laboratory and validated for their binding and biological activity in vitro and in vivo [32,33].

**Figure 1:**
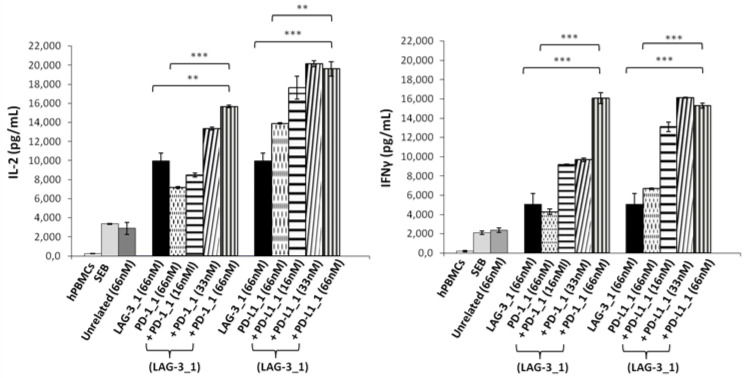
Effects of the combinatorial treatments on secretion of cytokines by stimulated T cells. hPBMCs were incubated with SEB (50 ng/mL) in the absence or presence of LAG-3_1, PD-1_1 or PD-L1_1 antibodies, used alone or in combinations for 66 h at 37 °C. Cytokine secretion was measured in the supernatants by evaluating the levels of IL-2 and IFNγ by ELISA. An unrelated antibody was used in parallel assays as a negative control. Error bars depict means ± SD. *p*-value: *** *p* ≤ 0.001; ** *p* < 0.01.

With this aim, human lymphocytes were treated with Staphylococcal enterotoxin B (SEB), and the mAbs specific for PD-L1 or PD-1, called PD-L1_1 or PD-1_1, respectively [32,33], were added at increasing concentrations in the absence or presence of a fixed concentration of LAG-3_1 (66 nM), and incubated for 66 h at 37 °C. The supernatants were then tested by ELISA assays to measure the secretion of IL-2 and IFNγ as markers of T cell activation. As shown in Figure 1, both the combinations of anti-PD-1 or anti-PD-L1 with LAG-3_1 mAb induced the secretion of cytokines more efficiently than the single agent treatments, as also reported in previous studies [32,35,36].

### 2.2. Construction of Novel Immunomodulatory Tribodies

On the basis of these promising results and the previous successful generation of antitumor tribodies [30,31], we decided to construct four different novel tribodies made of single chain variable fragments (scFv) or Fab chains derived from the parental LAG-3_1, PD-1_1 or PD-L1_1 mAbs.

**Figure 2:**
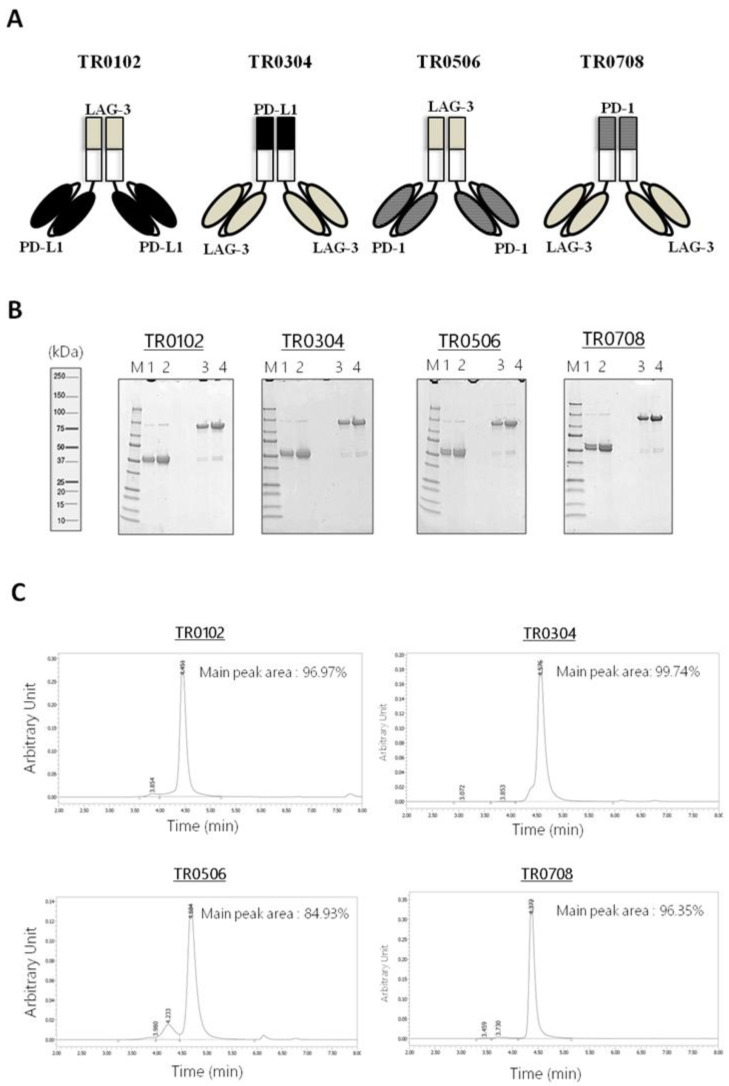
Schematic representation and purification of bispecific tribodies targeting LAG-3 and PD-L1 or PD-1, derived from LAG-3_1, PD-L1_1 and PD-1_1 parental mAbs. (**A**) Each TR was obtained by genetically fusing the indicated Fab with two identical scFvs. (**B**) Analysis of purity of the tribodies. Coomassie Blue stained SDS-PAGE of His-tagged Tribodies under reducing (lanes 1 and 2) or nonreducing conditions (lanes 3 and 4) obtained after purification by Ni-affinity chromatography. (**C**) SEC analysis of the final products to confirm the purity.

The novel tribodies, called TR0102, TR0304, TR0506 and TR0708, were obtained by exploiting the natural heterodimerization of the Fab derived either from LAG-3_1 antibody (for the TR0102 and TR0506), or the Fab of PD-L1_1 or PD-1_1 mAbs (for TR0304 and TR0708 respectively), fused with the two identical scFvs targeting a different IC, as shown in Figure 2A and listed in Table 1. Since the generated tribodies incorporated two different immune checkpoint binding domains, they were designed to combine the binding specificities and biological properties of two different immunomodulatory mAbs in a single molecule, and to achieve additive effects on T cell activation.

### 2.3. Purification and Analysis of the Stability of the Tribodies

Tribodies TR0102, TR0304, TR0506 and TR0708 were successfully expressed in Expi293 cells and purified from the cultured media by affinity chromatography followed by buffer exchange or gel filtration chromatography. The purity of the compounds was analyzed by SDS-PAGE (Figure 2B). Further size-exclusion chromatography (SEC) analyses were also performed to evaluate protein aggregation and/or stability after long-term storage (Figure 2C and Figure S1). As shown in Figure 2C, the tribodies were eluted in a single peak of the expected M.W. which represents the monomeric heterodimer from the H- and L-chain derivatives; only in the sample of TR0506 a higher M.W. Product was detectable at low levels (≤5–10%). The stability of TRs was also analyzed by SEC after storage at 4 °C for 4 weeks, as shown in Figure S1, as well as after three cycles of freeze and thaw, and no peak shape changes were observed in the novel constructs (data not shown).

### 2.4. Binding of the Novel Tribodies to Their Targets

**Figure 3:**
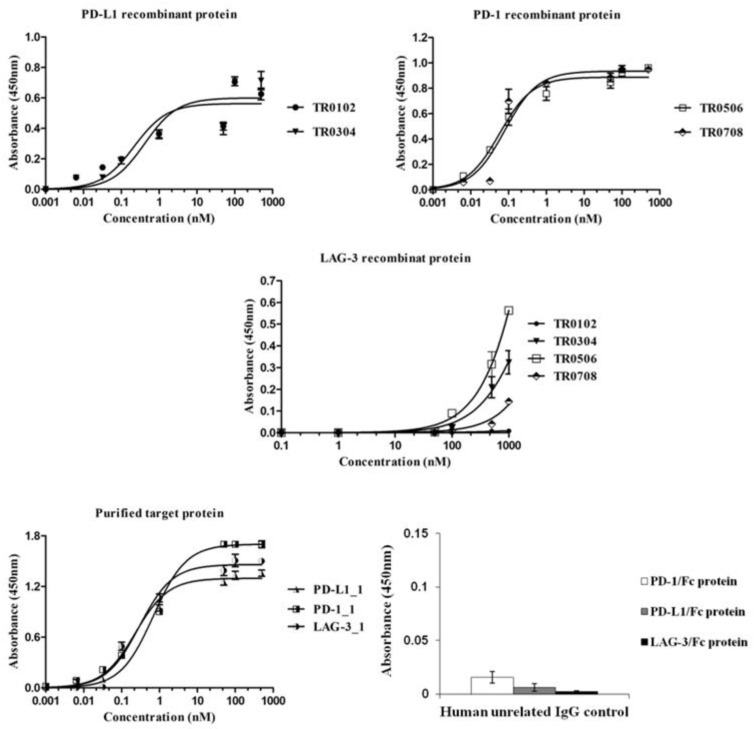
Binding of the novel generated tribodies to the purified recombinant proteins. Binding curves by ELISA assays of the tribodies (0.001–1000 nM), or their parental mAbs, onto immobilized PD-L1, PD-1 or LAG-3-Fc chimeric proteins. An unrelated human IgG antibody was used (at a concentration of 200 nM) in parallel assays as a negative control. The binding values were reported as the mean of at least three determinations obtained in three independent experiments. Error bars depict means ± SD.

**Figure 4:**
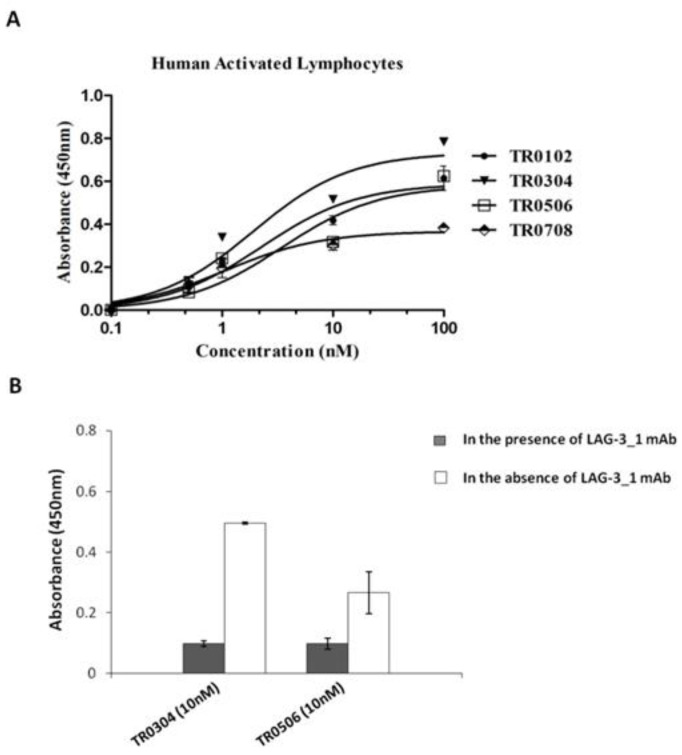
Binding affinity of the selected tribodies for activated lymphocytes in the absence or presence of LAG-3_1 mAb. (**A**) Cell ELISA assays were performed by using increasing concentrations (0.1–100 nM) of tribodies on activated hPBMCs. (**B**) Cell ELISA assays were performed by measuring the binding of the indicated tribodies to human-activated lymphocytes in the absence (empty bars) or presence (dark bars) of the parental anti-LAG-3 antibody used at saturating concentration. The binding values are reported as the mean of at least three determinations obtained in three independent experiments. Error bars depict means ± SD.

Once purified, the novel tribodies were tested by ELISA assays (Figure 3) for their ability to bind to the target recombinant proteins or to activated lymphocytes (Figure 4A) expressing the target ICs in their native conformation, in comparison with the parental mAbs. In parallel assays, a human unrelated isotype antibody was used at the highest concentration (200 nM) on all the three targets as a negative control.

All the indicated tribodies showed a binding affinity to purified PD-1 and PD-L1 proteins (Figure 3) in a sub-nanomolar range, and to activated lymphocytes (Figure 4A) in a low nanomolar range (Table 2). The Kd values obtained with PD-1 and PD-L1 for the tribodies were comparable to those of their parental mAbs, even though the maximum absorbance value was found to be lower. The binding affinity to purified LAG-3 protein appeared to be lower than that observed for the other two targets, and was limited to tribodies TR0304 and TR0506.

### 2.5. Evaluation of Tribody Interference in the Binding of ICs with Their Ligands

The unexpected low binding to LAG-3 protein can be explained by problems related to its low stability during the storage, assessed by monitoring (spectrofotometrically) its concentration and analyzing its degradation by SDS-PAGE over time. Thus, we decided to test the TR0304 and TR0506 TRBs on cells expressing LAG-3 in their native conformation. To confirm the binding specificity of the tribodies for LAG-3, we tested them on lymphocytes expressing high levels of LAG-3 in competitive ELISA assays with the parental anti-LAG-3 antibody. As shown in Figure 4B, the binding of tribodies TR0304 and TR0506 on human activated lymphocytes was significantly reduced in the presence of saturating concentrations (300 nM) of anti-LAG-3 mAb, indicating the specificity of these tribodies for LAG-3.

**Figure 5:**
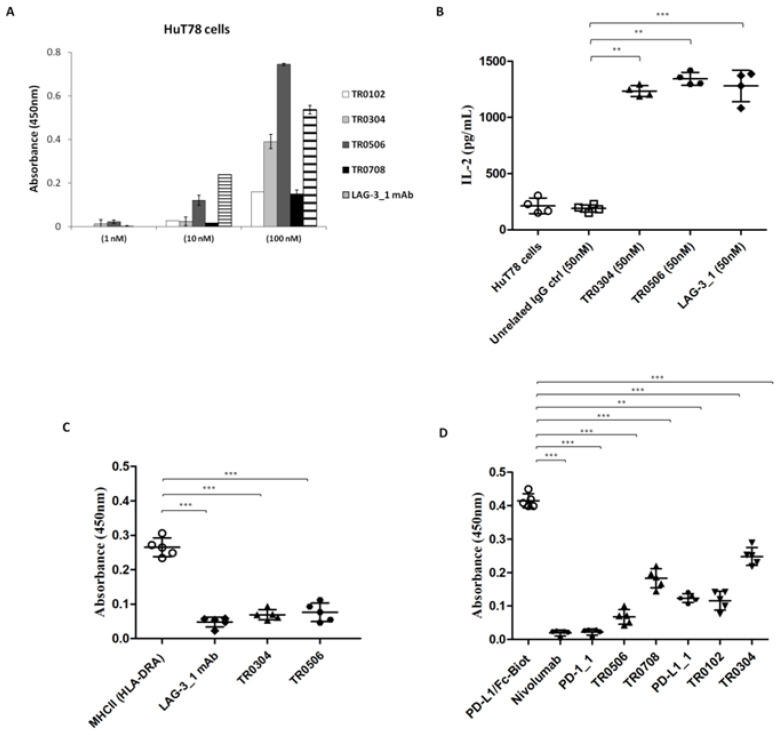
Binding specificity of the TRBs with target ICs and their antagonistic abilities. (**A**) Binding of the immunomodulatory tribodies, or LAG-3_1 parental mAb, tested at increasing concentrations on HuT78 cells by cell ELISA. Error bars depict means ± SD; these were calculated on the basis of results obtained by at least three independent experiments. (**B**) Secretion of IL-2 measured in the supernatants of HuT78 cells, treated as indicated, using a DuoSet ELISA kit. An unrelated antibody was used in parallel as a negative control. (**C**,**D**) Competitive ELISA to test the interference of the tribodies in LAG-3/MHCII (HLA-DRA) and PD-1/PD-L1 interactions. The binding of biotinylated MHCII or PD-L1 ligand to immobilized LAG-3 or PD-1 receptor, respectively, was measured in the absence or presence of a molar excess of tribodies (3:1 M/M for anti-LAG-3 and 5:1 M/M for anti-PD-L1 mAbs). Error bars depict means ± SD. *p*-value: *** *p* ≤ 0.001; ** *p* < 0.01.

To further confirm the binding specificity of the tribodies for LAG-3, we investigated their binding and biological effects on the HuT78 cell line, which is derived from CD4+ human T cell lymphoma and expresses high levels of LAG-3 receptor, but low or absent levels of PD-1 and PD-L1. In order to evaluate the ability of the tribodies to bind to these cells, we tested them at increasing concentrations, in parallel with the LAG-3_1 parental mAb, by cell ELISA. We found that the TR0304 and TR0506 tribodies bound to the cells with a comparable or even better specificity (Figure 5A). Thus, we also tested the ability of these tribodies to activate HuT78 cells. To this aim, we treated the cells with TR0304 and TR0506 at a concentration of 50 nM for 72 h. In parallel assays, the parental LAG-3_1 mAb was tested at the same concentration. The release of IL-2 was measured in the supernatants of the treated cells; the TR0304 and TR0506 tribodies were found to be able to significantly activate the cells (see Figure 5B) by inducing significant secretion of IL-2, i.e., comparable to that observed in the treatment with the parental LAG-3_1 mAb.

Furthermore, we analyzed the ability of these tribodies to interfere with LAG-3/MHCII (HLA-DRA) interactions by performing a competitive ELISA assay. To this end, LAG-3-His-GST recombinant protein was coated on plates at a concentration of 50 nM, TR0304 or TR0506 tribodies were added at a saturating concentration of 2 μM, and the binding of the Biotinylated-MHCII protein (700 nM) was measured. As shown in Figure 5C, the binding of biotinylated MHCII was strongly reduced in the presence of the tribodies or the parental LAG-3_1 mAb, used as a control, compared to the binding signal of the biotinylated MHCII used alone.

In parallel assays, to verify whether the novel human tribodies, derived from the fusion of LAG-3, PD-1 or PD-L1 binding domains, retained the ability of the parental mAbs to interfere in PD-1/PD-L1 interaction [21], we performed competitive ELISA assays by measuring the binding ability of biotinylated PD-L1 ligand to immobilized PD-1 receptor in the absence or presence of a molar excess (5:1 or 10:1 M/M) of TR0102, TR0304, TR0506 and TR0708 and compared these to biotinylated PD-L1 used alone. Nivolumab, PD-L1_1 and PD-1_1 parental mAbs were used in parallel assays as positive controls. As reported in Figure 5D, the binding of biotinylated PD-L1 ligand was significantly reduced in the presence of all the tribodies compared to biotinylated PD-L1 used alone, suggesting that the novel tribodies had retained the ability of the parental mAbs to interfere with interactions of PD-L1 with PD-1.

### 2.6. Effects of the Tribodies on the Activation of hPBMCs

**Figure 6:**
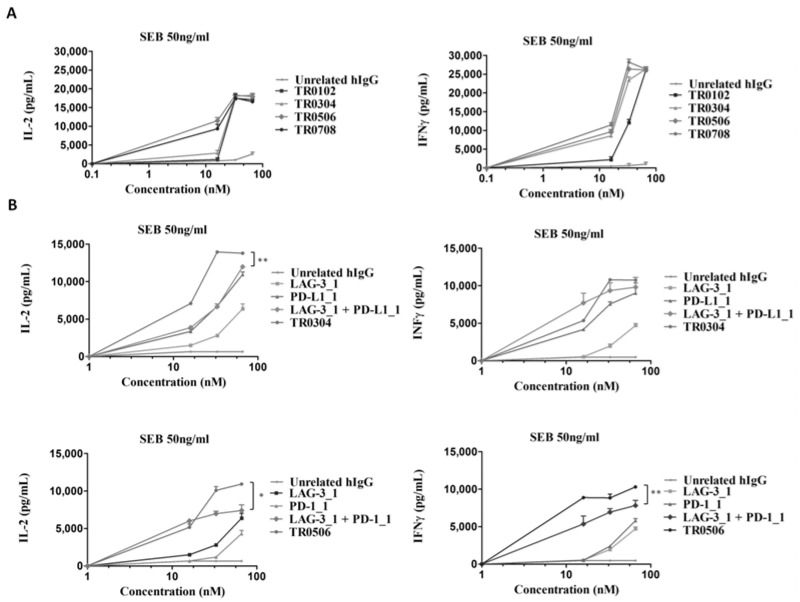
Effects of the tribodies on hPBMC activation and secretion of cytokines. The levels of IL-2 and IFNγ were measured by cytokine secretion assays on supernatants of hPBMCs stimulated with SEB (50 ng/mL) and treated with the indicated tribodies (**A**) or with TR0304 and TR0506 in comparison with their parental mAbs or combinations thereof (**B**) at the same concentrations. Cytokine concentration values are expressed in pg/mL. An unrelated antibody was used as a negative control. The values are reported as the mean of at least three determinations obtained in three independent experiments. Error bars depict means ± SD. *p*-value: ** *p* < 0.01; * *p* < 0.05.

To test whether the novel immunomodulatory tribodies were able to induce the activation of hPBMCs, we performed cytokine secretion assays on lymphocytes stimulated with SEB for 66 h and treated with increasing concentrations of each tribody. The levels of IL-2 and IFNγ released from T cells were measured by ELISA assays in the supernatants (Figure 6A). We found that the novel generated tribodies combined the functional properties of the different mAbs by activating lymphocytes with high efficacy; in particular, the EC50 values for the induction of IFNγ of TR0304, TR0506 and TR0708 were lower than that of TR0102. On the basis of these results and those relative to the higher specificity for LAG-3 of TR0304 and TR0506, we focused on these two tribodies for further functional characterization. To this end, the effects of these two tribodies on hPBMC activation compared to those of the parental immunomodulatory mAbs were analyzed by repeating the cytokine secretion assays. TR0304 and TR0506 were tested at increasing concentrations and the results were compared with those of the corresponding parental mAbs, used alone or in combination at the same concentrations. The results, reported in Figure 6B, show that TR0304 and TR0506 induced the secretion of cytokines from hPBMCs more efficiently than either the parental mAbs or their respective combinations, thus confirming the advantage of fusing two immunomodulatory moieties into a single tribody to achieve more potent activation of immune cells.

### 2.7. In Vitro Antitumor Effects of the Tribodies on Breast Cancer Cells

**Figure 7:**
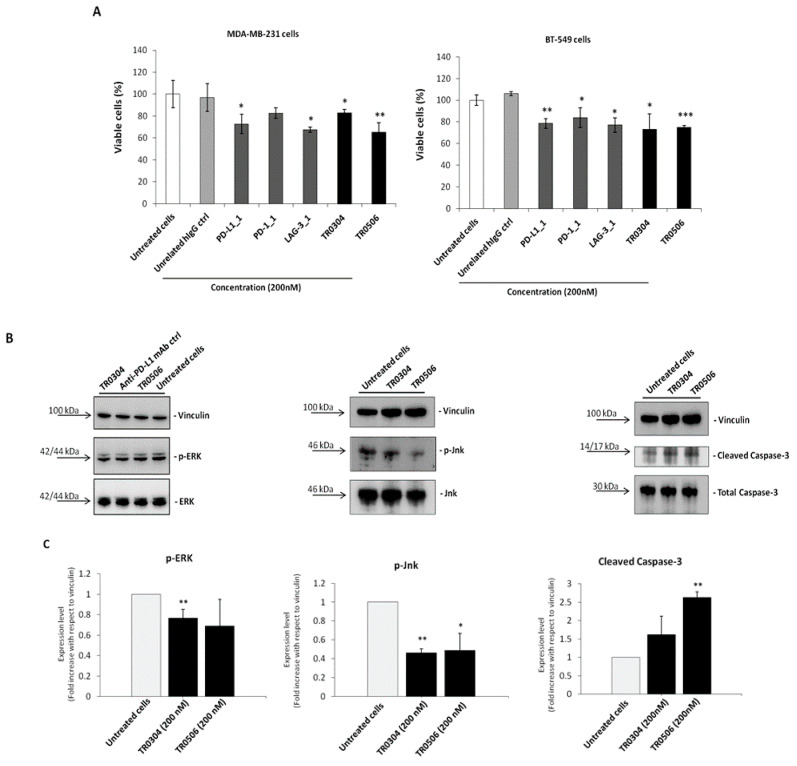
The novel tribodies inhibit tumor cell viability by affecting intracellular pathways. (**A**) MDA-MB-231 and BT-549 tumor cells were treated for 72 h with the tribodies TR0304, TR0506, the parental PD-L1_1, PD1.1 and LAG3.1 mAbs or a human unrelated IgG at the concentration of 200 nM. Cells were counted by using the Trypan Blue exclusion test and cell survival is expressed as percentage of viable cells with respect to the untreated cells. (**B**) Western blotting analyses of ex-tracts from MDA-MB-231 breast tumor cells, treated for 72 h as indicated, by using the anti-Erk or -phospho-Erk, the anti-Jnk or -phospho-Jnk, the anti-Caspase-3 or anti-Cleaved Caspase3 an-tibodies and the anti-vinculin Ab, used to normalize. (**C**) Densitometry quantification of signals from Western blotting analyses. The protein levels of phospho-Erk, phospho-Jnk or Cleaved Caspase-3 are expressed as fold increase with respect to cells untreated and the intensity of the bands was normalized to vinculin. The values were reported as the mean of at least three determinations obtained in three independent experiments. Error bars depicted means ± SD. *p*-value: *** *p* ≤ 0.001; ** *p* < 0.01; * *p* < 0.05.

Since PD-1, PD-L1 and LAG-3 were also found to be expressed on different types of tumor cells, such as breast cancer cells [33,35,36], and the parental mAbs PD-L1_1 and PD-1_1 have previously shown the ability to bind to IC-positive tumor cells by inhibiting their growth, even in the absence of immune cells, we investigated the effects of the novel tribodies on tumor cell viability in the absence of lymphocytes. To this end, MDA-MB-231 and BT-549 triple negative breast tumor cells, expressing satisfactory levels of PD-L1 and PD-1 and moderate levels of LAG-3 [35,36], were plated in 96-well plates and treated with the tribodies, or their corresponding parental mAbs, at a concentration of 200 nM for 72 h. As shown in Figure 7A, the novel bispecific tribodies slightly inhibited tumor growth, i.e., by about 20–35%, even in the absence of immune cells, in a similar fashion to the parental mAbs, thus confirming that they had retained their antitumor activity.

To verify whether the tribodies affected the survival pathways previously identified for their parental mAbs [33,35,36], we performed Western Blotting analyses of protein extracts from MDA-MB-231 tumor cells treated for 72 h at 37 °C in the absence or presence of the tribodies, or the parental PD-L1_1 mAb, used at a concentration of 200 nM. As shown in Figure 7B, the levels of phosphorylated Erk and Jnk proteins decreased when the cells were treated with TR0304 and TR0506, whereas the levels of Cleaved Caspase-3 increased after treatments, confirming the involvement of the same intracellular pathways downstream of PD-L1 and PD-1, affected by their parental mAbs [33].

### 2.8. In Vitro Cytotoxic Effects of Tribodies on Breast Tumor Cells Co-Cultured with Human Lymphocytes

**Figure 8:**
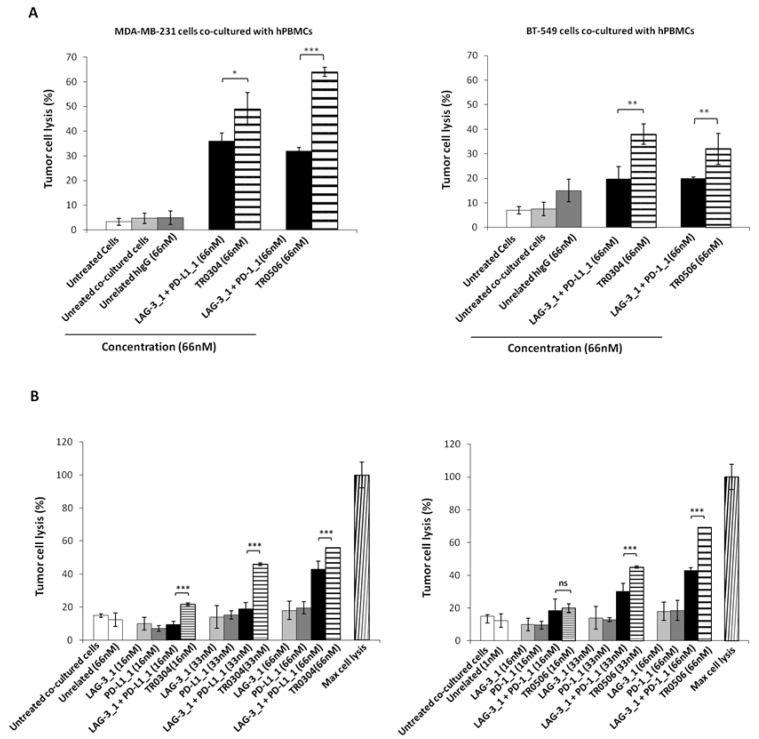
Cytotoxic effects of tribodies or their parental mAbs on breast tumor cells co-cultured with human lymphocytes. (**A**) Tumor cells lysis was evaluated by measuring the levels of LDH released in the supernatants of MDA-MB-231 (left panel), or BT-549 (right panel) cells cocultured with hPBMCs (6:1 Effector:Target ratio), treated as indicated for 48 h. (**B**) MDA-MB-231 cells co-cultured with hPBMCs (6:1 E:T ratio) were treated with the TR0304 and TR0506 tribodies or the corresponding parental mAbs at increasing concentrations for 48 h, and the levels of LDH in the supernatants were measured and expressed as % of maximum cell lysis. Maximum cell lysis was obtained by adding 0.9% Triton X-100 (Thermo Fisher Scientific, Darmstadt, Germany). Error bars depict means ± SD. *p*-value: *** *p* ≤ 0.001; ** *p* < 0.01; * *p* < 0.05.

Since the novel tribodies targeting PD-L1, PD-1 and LAG-3 efficiently induced the activation of lymphocytes, we further investigated their antitumor effects on co-cultures of tumor cells and lymphocytes. To this end, MDA-MB-231 and BT-549 breast cancer cells expressing high levels of PD-L1 [36] were cocultured with hPBMCs in the absence or presence of the TR0304 and TR0506 tribodies at a concentration of 66 nM for 48 h at 37 °C. In parallel assays, we tested the parental mAbs, used in combination at the same concentrations. After incubation, cells lysis was measured by lactate dehydrogenase (LDH) released in the cell supernatants [37]. The results showed that the tribodies induced tumor cells lysis with higher efficacy than the parental mAbs or combinations thereof (see Figure 8A and Figure S2).

Considering the highest antitumor efficacy obtained with the TR0304 and TR0506 tribodies, we investigated the dose dependent effects of these two immunomodulatory tribodies in comparison with the parental mAbs or combinations thereof used at the same concentrations.

To this end, the indicated tribodies or the corresponding parental mAbs were added to the co-cultures at increasing concentrations, and the cells were incubated at 37 °C for 48 h. Cells lysis was measured by LDH assays on cell supernatants. The results showed that the TR0304 and TR0506 tribodies (see Figure 8B) induced tumor cell lysis with higher efficacy than the parental mAbs when used in combinatorial treatments at the same concentrations, thus confirming the beneficial effects of combining two different immunomodulatory mAbs into a single molecule.

### 2.9. Analysis of Possible Side Effects of TRs on Circulating Naive Lymphocytes

**Figure 9:**
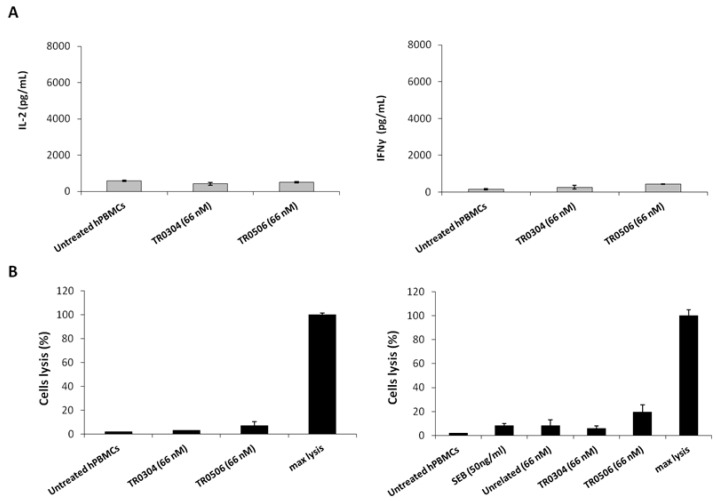
Effects of tribodies on naive lymphocytes. (**A**) Cytokines assays were performed on lymphocytes by treating them with the tribodies in the absence of stimuli. IL-2 and IFNγ released by lymphocytes were quantified and expressed as pg/mL. (**B**) To confirm the lack of toxicity of immunomodulatory tribodies on lymphocytes, T cell lysis was analyzed by measuring the increase of LDH in the supernatants of unstimulated hPBMCs treated with the tribodies, as indicated. Untreated hPBMCs were used as a negative control. Error bars depict means ± SD.

To rule out the possibility of nonspecific side effects on circulating naive lymphocytes, the novel tribodies were incubated with unstimulated lymphocytes for 66 h. The levels of IL-2 and IFNγ cytokines released from unstimulated lymphocytes treated with tribodies were found to be very low (Figure 9A), proving that the effects of the tribodies were exerted only on activated lymphocytes expressing ICs. Similarly, to exclude the possibility that the tribodies induced lysis of T cells, cytotoxic assays were performed on lymphocytes untreated or treated with the tribodies by evaluating the release of LDH from lymphocytes in the supernatants. As shown in Figure 9B, the tribodies did not show significant effects on the lysis of lymphocytes.

### 2.10. Comparison of the Cardiotoxic Side Effects of Novel Tribodies with Those of the Respective Parental Compounds Used in Combination

Despite the approval of several different ICIs used in combination against a variety of targets with complementary mechanisms of action, severe immune-related adverse events (irAEs), such as myocarditis, can occur in some patients [38,39].

**Figure 10:**
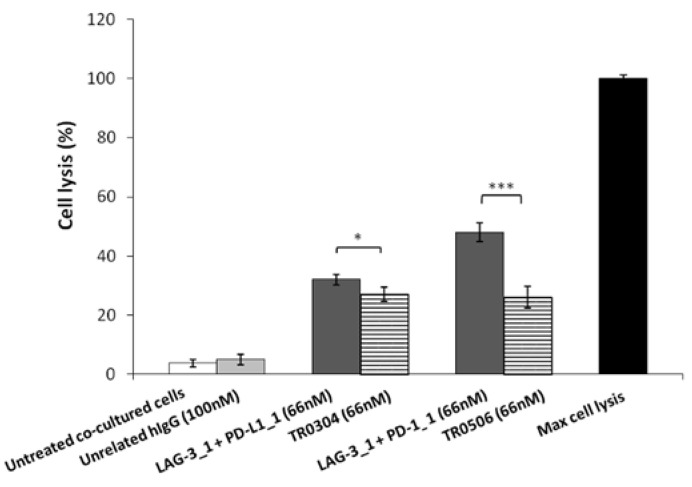
Cardiotoxic effects induced by immunomodulatory tribodies or the combination of their respective parental compounds on human fetal cardiomyocytes. The lysis of HFC cells, co-cultured with hPBMCs in the absence (white bars) or presence of tribodies (striped bars) or the combinations of their respective compounds (grey bars) for 24 h, was analyzed by measuring the release of LDH in the supernatants of cultures. The values are reported as the mean of at least three determinations obtained in three independent experiments. Error bars depict means ± SD. *p*-value: *** *p* ≤ 0.001; * *p* < 0.05.

Thus, to evaluate the safety of the novel tribodies, we investigated their effects on Human Fetal Cardiomyocytes (HFC) co-cultured with hPBMCs, and compared their possible cardiac side effects with those of the combination of their respective parental compounds. To this end, HFC were co-cultured with hPBMCs (Effector:Target ratio 5:1) and treated with TR0304 and TR0506 or the combination of their parental mAbs (LAG-3_1 + PD-L1_1 or LAG-3_1 + PD-1_1, respectively) for 24 h at a concentration of 66 nM. Untreated cells or cells treated with an unrelated human IgG1 mAb were used as controls. The lysis of cardiac cells was evaluated by measuring the level of LDH released in the supernatants of cell cultures after the indicated treatments. As reported in Figure 10, the combination of PD-L1_1 or PD-1_1 with LAG-3_1 mAbs induced higher lysis (about 40%) of cardiac cells compared to their corresponding TR0304 or TR0506 tribody constructs (about 20%).

These results indicate that the novel tribodies showed higher in vitro antitumor efficacy and lower potential cardiotoxic side effects than combinatorial treatments of the parental mAbs, indicating that advantages, in terms of safety, can be obtained by using a single bispecific construct, such as a tribody, compared to combinations of different mAbs.

## 3. Discussion

In this study, we investigated novel combinations of mAbs targeting LAG-3, PD-L1 or PD-1, previously generated in our laboratory and validated for biological activity in vitro and in vivo. We identified the most efficient combinations of these mAbs by testing their effects on the activation of T cells. The leading candidates were then used for the construction of four novel human tribodies, called TR0102, TR0304, TR0506 and TR0708, made up of two single chain variable fragments (scFv) and a Fab derived from the parental LAG-3_1, PD-1_1 or PD-L1_1 mAbs. In particular, TR0102 and TR0506 were made up of the Fab derived from LAG-3_1 antibody, fused with two identical scFvs targeting PD-L1 or PD-1, respectively, whereas TR0304 and TR0708 were constructed by using the Fab of PD-L1_1 or PD-1_1 mAbs, respectively, fused with two identical scFvs derived from LAG-3_1 targeting LAG-3.

The binding of these novel tribodies was evaluated on both activated T cells, expressing the targets in their native conformation, and on purified recombinant targets. All the tribodies retained the binding ability of the corresponding parental anti-PD-1 and anti-PD-L1 mAbs with an affinity in the nanomolar range. The affinity of the tribodies for LAG-3 was found to be lower in the purified protein but satisfactory on activated hPBMCs and on cell lines, such as the T cell lymphoma HuT78, expressing high levels of LAG-3 and low levels of PD-1 and PD-L1.

More interestingly, the novel tribodies were also found to be able to interfere with receptor-ligand interactions (PD-1/PD-L1, MHCII/LAG-3), thus confirming that the antagonistic properties of the parental mAbs were preserved in the novel constructs.

When tested on activated lymphocytes, the tribodies were found to induce a more efficient activation of hPBMCs compared to the combination of the parental mAbs. On the basis of these results, the advantage of fusing two moieties targeting different ICs into a single tribody to achieve more potent activation of immune cells is clear. To further confirm these promising findings, we tested the novel bispecific constructs in co-cultures of tumor cells and lymphocytes. The novel tribodies TR0304 and TR0506 were found to be able to induce dose dependent tumor cell lysis by showing higher cytotoxic effects than the parental antibodies or combinations thereof, thus confirming the advantages of combining two different immunomodulatory mAbs into a single construct to increase the antitumor potency. Furthermore, since the parental mAbs were found to be able to activate both stimulated CD4^+^ and CD8^+^ T cells [32] in a similar manner, we expect that the derived tribodies could also be used to induce immune responses by both immune cell populations.

Finally, the tribodies were also studied to verify whether they exerted off-target toxicity on circulating naive lymphocytes that could lead to unwanted side effects. To this end, unstimulated lymphocytes in the absence of tumor cells were treated with the tribodies, and secretion of cytokines and LDH release were tested and found to be very low, suggesting that the tribodies neither activated naïve T cells not expressing high levels of ICs, nor elicited off target cytokine release.

Recent studies have reported that a fraction of patients treated with combinations of ICI have an increased risk of severe immune-related adverse events such as myocarditis, a severe cardiac complication, due to the expression of some ICs, such as PD-1 and PD-L1 [40,41], in the myocardium.

Thus, to investigate the possible cardiotoxic effects of the novel tribodies, we tested their effects on co-cultures of cardiomyocytes with lymphocytes. The novel tribodies induced lower cardiotoxicity than the parental mAbs or their combinations, indicating that advantages in terms of safety can be obtained by using a single bispecific construct, such as a tribody, compared to combinations of mAbs.

The novel tribodies could have some advantages with respect to BiTEs, such as: the ability to bind to three different targets [30,42]; higher molecular weight, that could achieve longer half-life in circulation, as previously reported for other tribodies [43]; and Fc-free format, that avoids the induction of nonspecific cytokine storms of different bispecific molecules including Fc, such as Catumaxomab, an anti-CD3/EpCAM used for the intraperitoneal treatment of malignant ascites. Other bispecific mAbs in clinical use or trials are: Cinrebafusp alfa (PRS-343), a bispecific fusion protein targeting both HER2/4-1BB on tumor cells and T cells, currently in phase I of clinical trial (NCT03330561) for HER2-positive tumor patients [14,15,16]; and KN046, an IgG1 bsAb targeting both PD-L1 and CTLA-4, currently in a phase I clinical trial (NCT03529526) on metastatic triple negative breast cancer [17,18,19]. However, both of these contain the Fc portion, and thus, could have higher nonspecific side effects and larger molecular weights than the novel tribodies.

Thus, we can conclude that the novel tribodies have the potential to become effective and safe therapeutic drugs for increasing T cell infiltration in resistant tumors.

## 4. Materials and Methods

### 4.1. Cell Cultures

MDA-MB-231 breast cancer cells were cultured in Dulbecco’s Modified Eagle’s Medium (DMEM, Gibco, Life Technologies, Paisley, UK). BT-549 breast cancer cells were cultured in Roswell Park Memorial Institute 1640 Medium (RPMI 1640, Gibco, Life Technologies, Paisley, UK). HFC human fetal cardiomyocytes were cultured in Cardiac Myocyte Medium (CMM, Innoprot, DeriBizkaia, Spain), according to the manufacturer’s recommendations. The media were supplemented with 10% (vol/vol) heat-inactivated fetal bovine serum (FBS, Sigma, St. Louis, MO, USA), and were used after addition of 50 U/mL penicillin, 50 μg/mL streptomycin, 2 nM L-glutamine (all from Gibco, Life Technologies, Paisley, UK). The HuT78 cutaneous T lymphocyte cell line, from ATCC, was cultured in Iscove’s Modified Dulbecco’s Medium (IMDM, Sigma-Aldrich, St. Louis, MO, USA), supplemented with 20% (vol/vol) FBS, 50 U/mL penicillin, 50 μg/mL streptomycin, 2 nM L-glutamine. All cell lines were cultured in a humidified atmosphere containing 5% CO_2_ at 37 °C.

### 4.2. Antibodies and Human Recombinant Proteins

The following recombinant proteins were used: Human PD-L1/Fc, human PD-1/Fc and human LAG-3/Fc protein (all from Bio-Thecne R&D Systems, Inc., Minneapolis, MN, USA); Human LAG-3/His-GST and Human HLA class II histocompatibility antigen, DRA (from Cusabio Technology LLC, Houston, TX, USA); and Staphylococcal enterotoxin B (SEB), a toxin produced by the bacterium Staphylococcus aureus and used as stimuli for the activation of lymphocytes (Sigma, S4881 St. Louis, MO, USA).

The following antibodies were used: HRP-conjugated antihuman IgG (Fab’)2 goat polyclonal antibody (Abcam, Milan, Italy); anti-His-HRP-conjugated antibody (Proteintech, Deansgate, Germany); HRP-conjugated Streptavidin (Biorad, Milan, Italy); antihuman p44/42 MAPK (T202/Y204), antihuman Cleaved Caspase-3; anti-phospho-Sapk/Jnk (T183/Y185) rabbit polyclonal antibodies (all from Cell Signaling, Danvers, MA, USA); antivinculin monoclonal antibody (all from Santa Cruz Biotechnology, Inc. Dallas, TX, USA); and HRP-conjugate anti-mouse IgG and antirabbit secondary antibodies (all from Sigma, USA). PD-L1_1 (anti-PD-L1), PD-1_1 (anti-PD-1), LAG-3_1 (anti-LAG-3). Human IgG control (unrelated) monoclonal antibodies were produced in our laboratory, as previously described [32].

### 4.3. Production and Purification of Tribodies

The tribodies indicated in Table 1 were produced by Chiome, Tokyo, Japan by using the Trisoma platform technology to express and purify the constructs. The nucleic acid sequences encoding anti-PD-1, anti-PD-L1 and anti-LAG-3 scFvs or Fabs, used for the construction of tribodies by the insertion of classical (Gly_4_-Ser)_n_ linkers, were obtained from antibodies that specifically bind to PD-1, PD-L1 and LAG-3, as described in patent PCT/EP2019/057239, and previously published as WO2019180201A2. Sequence analyses of the final tribodies confirmed that no mutations or errors had been introduced. Briefly, Expi293 cells were cotransfected with respective expression vectors of heavy chain derivative and light chain derivative corresponding to TR0102, TR0304, TR0506 and TR0708, using Expifectamine as the reagent, according to manufacturer’s instructions (Thermo Fisher Scientific, Darmstadt, Germany). Five days post-transfection, tribodies were purified by affinity chromatography with complete His-tag purification resin (Sigma St. Louis, MO, USA), followed by buffer exchange or gel filtration chromatography. This process recovers the tribody while limiting impurities such as product variants. The purified tribodies were analyzed by SDS-PAGE and size-exclusion chromatography (SEC) analysis to evaluate protein aggregation and stability. A > 95% L/Fd chain tribody monomer was confirmed. Tribodies were then sterile filtered, aliquoted and stored at −80 °C.

### 4.4. Cytotoxicity and Cell Viability Assays

To evaluate the effects of the tribodies on tumor cell growth, MDA-MB-231 cells were plated at a density of 1 × 10^4^ cells/well in 96-well flat-bottom plates, and incubated for 16 h at 37 °C. The tribodies were added at a concentration of 200 nM in the complete culture medium and incubated for 72 h. Viable cells were counted by trypan blue exclusion test, and cell survival was expressed as the percentage of viable cells in the presence of the drugs being tested with respect to negative control cultures grown in the absence of such treatments.

To measure the cytotoxic effects of the novel immunomodulatory tribodies on cocultures, target tumor cells or human cardiomiocytes were incubated with hPBMCs (effector: target ratio 6:1) and treated with the tribodies at different concentrations, or with the parental mAbs and their corresponding combinations. To this end, tumor cells were plated in 96-well flat-bottom plates at a density of 1 × 10^4^ cells/well for 16 h. Then, hPBMCs isolated from healthy donors were added in the absence or presence of the treatments, and the cells were incubated at 37 °C for 48 h. Untreated cells or cells treated with an unrelated IgG were used as negative controls. The lysis of the target cells was measured by detecting the levels of LDH released in the supernatant of the cocultures, using the LDH detection kit (Thermofisher Scientific, Rockford, IL, USA), following the manufacturer’s recommendations. Cell lysis was analyzed by measuring the fold increase of LDH in the presence of each treatment with respect to the amount present in the supernatant of cocultures untreated or treated with the unrelated antibodies and expressed as percentage with respect to the maximum lysis obtained by adding 0.9% Triton X-100 (Thermo Fisher Scientific Darmstadt, Germany).

### 4.5. Cytokine Secretion Assays

The secretion of human IL-2 and IFNγ in the supernatants of hPBMCs, unstimulated or stimulated with SEB and treated with the tribodies or their corresponding parental mAbs used alone or in combination, was measured by using DuoSet ELISA kit (Bio-Thecne R&D Systems Inc., Minneapolis, MN, USA) assays. The levels of IL-2 were also detected in the supernatant of HuT78 cells untreated or treated with the tribodies in order to investigate their effects on a human T cell lymphoma-derived cell line. Briefly, after treatments, culture supernatants were centrifuged and the cytokines quantified using IL-2 and IFNγ kits (from R&D Systems, Minneapolis, MN, USA), according to the producer’s recommendations. Concentration values were converted in pg/mL and are reported as the mean of at least three determinations.

### 4.6. ELISA Assays

#### 4.6.1. Binding Assays

To confirm the binding specificity of the novel generated tribodies, ELISA assays were performed on human chimeric target proteins (PD-L1/Fc, PD-1/Fc or LAG-3/Fc coated at 5 μg/mL on NUNC 96-well plates) and on human activated hPBMCs or HuT78 cells (3 × 10^5^ for each well in round 96-well plate).

The ELISA assays on coated chimeric protein were performed by blocking the coated plates with 5% nonfat dry milk in PBS for 1 h at 37 °C. The purified compounds were added to the plates at different concentrations in 3% BSA (bovine serum albumin) in PBS 1X and incubated for about 90 min at room temperature with gentle shaking. After extensive washes with PBS, the plates were incubated with HRP-conjugated antihuman IgG (Fab’)2 goat polyclonal antibody (Abcam, Milan, Italy) or anti-His-HRP-conjugated antibody (Proteintech, Deansgate, Germany) for 1 h, washed again and incubated with TMB reagent (Sigma, St. Loius, MO, USA) for 10 min before quenching with an equal volume of 1 N HCl.

Cell ELISA assays were performed by incubating the lymphocytes after stimulation with SEB (50 ng/mL for 72 h) or HuT78 cells after preincubation with saturating concentrations of LAG-3_1 mAb, with increasing concentrations of the purified compounds in 3% BSA in PBS 1X for about 90 min at room temperature with gentle agitation. Plates were then centrifuged, and cell pellets were washed with PBS and incubated with the secondary antibodies, as mentioned above. The absorbance at 450 nm was measured using an Envision plate reader (Perkin Elmer 2102 Waltham, MA, USA).

The Kd values were calculated by elaboration of ELISA binding curve analyses obtained with activated human lymphocytes using the Prism (GraphPad) tool according to the following model: Y = Bmax × X/(Kd + X) + NS × X + Background.

#### 4.6.2. Competitive Assays

In order to investigate the ability of the novel generated tribodies, in comparison with the corresponding parental mAbs, to compete for PD-L1/PD-1 or LAG-3/MHC II (HLA-DRA) binding, competitive ELISA assays were performed by testing the binding of each biotinylated chimeric recombinant protein (PD-L1/Fc or MHCII) to immobilized PD-1 or to LAG-3/GST-His in the absence or presence of the unlabeled competitive tribodies. To this end, the coated plates were pre-incubated with the competitor tribodies or their corresponding parental mAbs for 2 h at room temperature (at 5:1 M/M excess ratio for anti-PD-L1 or -PD-1, and 3:1 for LAG-3), and then further treated with the biotinylated proteins, which were added to the plate at the same concentrations as the competitive antibodies for 2 h at room temperature. To detect the binding of the biotinylated proteins, HRP-conjugated Streptavidin was added and the same subsequent steps were performed as described above.

### 4.7. Western Blotting Analysis of Cell Extracts

MDA-MB-231 cells grown in six-well plates at 37 °C and treated with the tribodies at a concentration of 200 nM for 72 h were collected and lysed, as previously reported [44]. Protein concentrations were determined by Bradford colorimetric assay (Biorad, California, USA), and Western blotting analyses were performed by incubating the membranes with the anti-phospho-p44/42 MAPK, anti-p44/42 MAPK, anti-phospho-Sapk/Jnk, anti-Sapk/Jnk, anti-cleaved Caspase-3, anti-Caspase 3 and anti-vinculin antibodies (Cell Signaling Technology, Danvers, MA, USA), followed by incubation with the appropriate HRP-conjugated secondary antibodies and detection, as previously reported [45].

### 4.8. Statistical Analyses

Error bars were calculated on the basis of the results obtained by at least three independent experiments. Statistical analyses were assessed by Student’s *t*-test (two variables). Statistical significance was established as *** *p* ≤ 0.001; ** *p* < 0.01; * *p* < 0.05.

## 5. Conclusions

We provide evidence that novel human immunomodulatory bispecific tribodies are endowed with more potent antitumor efficacy than parental compounds when used either as single agents or in combination. Interestingly, no significant increase in side effects was observed. Thus, we confirm the advantages of combining two immunomodulatory mAbs into a single construct to obtain effective and safe therapeutic drugs.

## Figures and Tables

**Table 1:** List of the novel generated tribodies and their composition of scFvs and Fabs derived from immunomodulatory mAbs.

Protein ID	Sequence
**TR0102**	(Fab)LAG-3-(scFv)2xPD-L1
**TR0304**	(Fab)PD-L1-(scFv)2xLAG-3
**TR0506**	(Fab)LAG-3-(scFv)2xPD-1
**TR0708**	(Fab)PD-1-(scFv)2xLAG-3

**Table 2:** Kd values obtained from the binding curves of the tribodies to activated hPBMCs.

Kd	TR0102	TR0304	TR0506	TR0708
hPBMCs	2.265 nM	1.910 nM	3.439 nM	1.057 nM

## Supplementary Materials

The following supporting information can be downloaded at: https://www.mdpi.com/article/10.3390/ijms23073466/s1.
