# Supplementary material for: Novel Bi-Specific Immuno-Modulatory Tribodies Potentiate T Cell Activation and Increase Anti-Tumor Efficacy
Source: Int J Mol Sci. 2022 Mar 23;23(7):3466. doi: 10.3390/ijms23073466 (PMC8998846; doi:10.3390/ijms23073466)
Supplement: Supplementary file 1 [file ijms-23-03466-s001.zip › ijms-1638293-supplementary.pdf]

Novel Bi-Specific Immuno-Modulatory Tribodies Potentiate T Cell Activation And Increase Anti-Tumor Efficacy

Margherita Passariello[1,2], Asami Yoshioka[3], Kota Takahashi[3], Shu-ichi Hashimoto[3], Rosa Rapuano Lembo[2,4], Lorenzo Manna[1,2], Koji Nakamura[3] and Claudia De Lorenzo[1,2*]

1 Department of Molecular Medicine and Medical Biotechnology, University of Naples “Federico II”, 80131 Naples, Italy

2 Ceinge - Biotecnologie Avanzate s.c. a.r.l., via Gaetano Salvatore 486, 80145 Naples, Italy

3 Chiome Bioscience Inc, 3-12-1 Hommachi Shibuya-Ku, Tokyo 151-0071 Japan

4 European School of Molecular Medicine, University of Milan, 20122 Milan, Italy

* Correspondence: cladelor@unina.it; Tel.: +39-081-3737868

Supplementary Figures and Legends

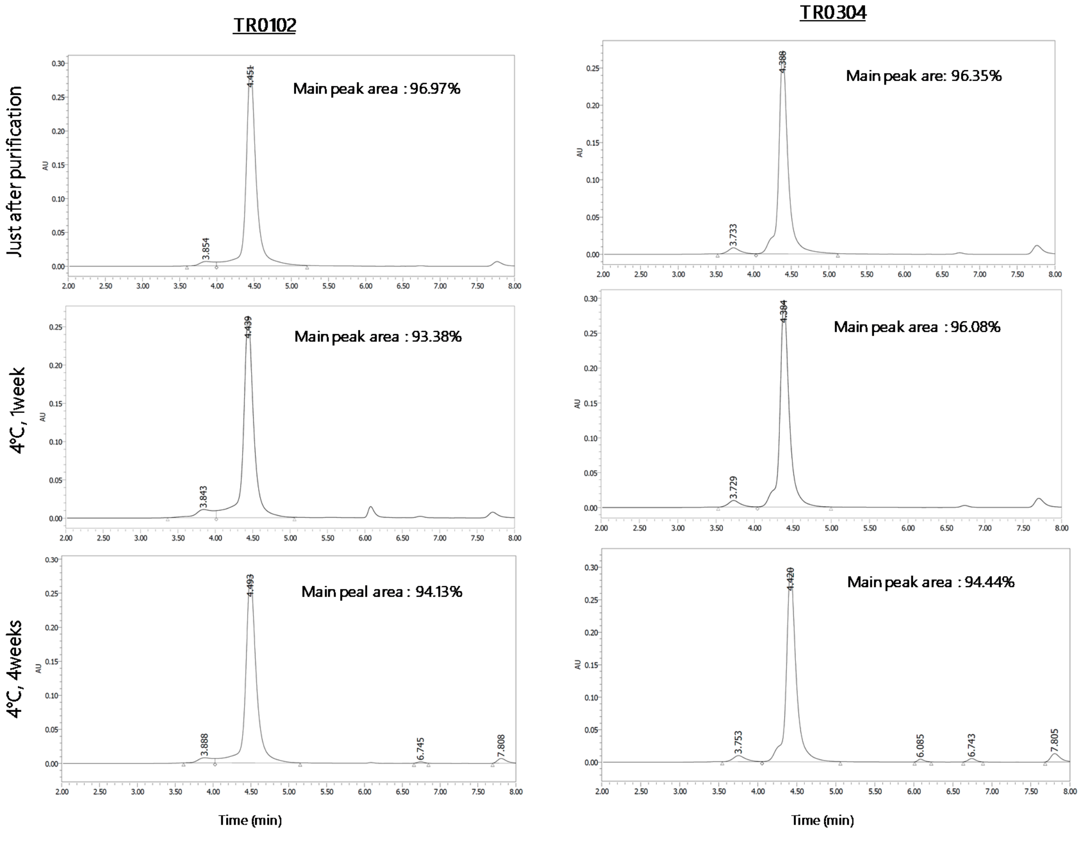

Figure S1. SEC Analysis. The analysis of the purified samples to verify the stability of tribodies was also performed after storage at 4°C for up to 4 weeks.

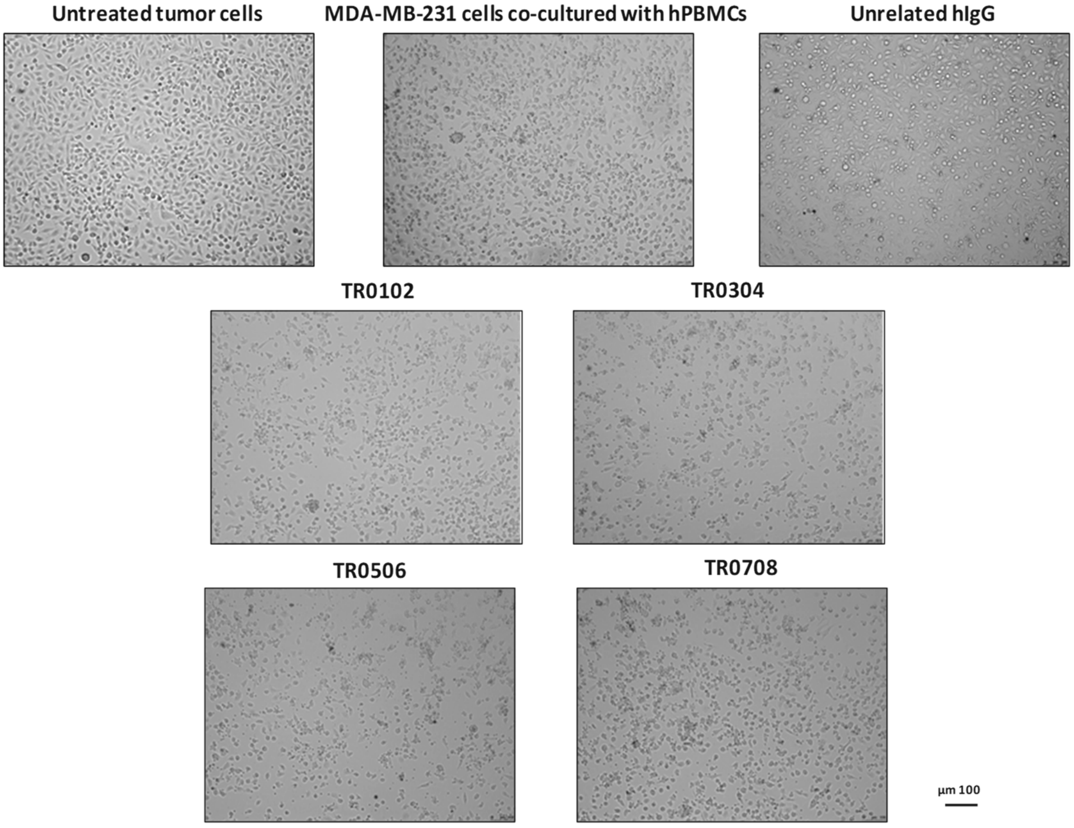

Figure S2. Representative images of MDA-MB-231 cells co-cultured with hPBMCs and treated with the indicated tribodies**.**
